# The application of 6S and PDCA management strategies in the nursing of COVID-19 patients

**DOI:** 10.1186/s13054-020-03124-w

**Published:** 2020-07-16

**Authors:** Wenju Wei, Sicong Wang, Hongliang Wang, Hongjia Quan

**Affiliations:** 1 Department of Critical Care Medicine, The Second Affiliated Hospital of Harbin Medical University, No. 246, Xuefu Road, Nangang District, Harbin, 150001 Heilongjiang China; 2 Heilongjiang Province Medical Aid Group for CVOID-19, Wuhan, Hubei China; 3 Department of Critical Care Medicine, Cancer Hospital of Harbin Medical University, Harbin, Heilongjiang China

At the end of 2019, the coronavirus disease 2019 (COVID-19) [1] spreads across the world [2]. Hubei Province has been one of the most affected areas in China. In response to the national call for medical personnel, the medical assistance team of Heilongjiang Province arrived at Wuhan Union Hospital right away.

6S [3] is a management model for enterprise operation that is derived from the 5S concept proposed by Hiroyuki Hirano [4]. The core of 5S includes Seiri, Seiton, Seiso, Seiketsu and Shitsuke. Years of practice have proven that 5S can significantly improve the efficiency and quality of corporate work, enhance corporate image and create a good working atmosphere. 6S adds the concept of security to the 5S model. While helping at the hospital, the nursing assistance team of Heilongjiang Province fully applied the concept of 6S to manage the team and achieved good results, such as zero infections among medical staff, zero nursing accidents and zero complaints from patients. The experience is summarized in this paper in the hope of helping in the global fight against the epidemic.

## Challenges in nursing

As the medical assistance team was the first to arrive, no precedents had been set; thus, the following problems were encountered at the initial stage of their work:

1. The Heilongjiang nursing aid team in Hubei comprised members from diverse departments of numerous hospitals in the province, including members of critical care medicine, respiratory, infection, cardiovascular and other departments. Therefore, the employees had different working habits and skills, which required some integration and task redistribution.
2. There were differences in the working habits and dialects of the local medical staff and the supporting medical staff.
3. At the initial stage of the epidemic, medical supplies were in relatively short supply; therefore, it became a major challenge to rationally manage and distribute the protective and treatment supplies and to ensure the safety of medical staff.
4. The supporting time was during Chinese New Year, and the team members generally had feelings of loneliness and fear. Under such circumstances, it was necessary to closely monitor the mental state of the team members while also ensuring their physical health.

## Application of the 6S management mode

**Fig. 1:**
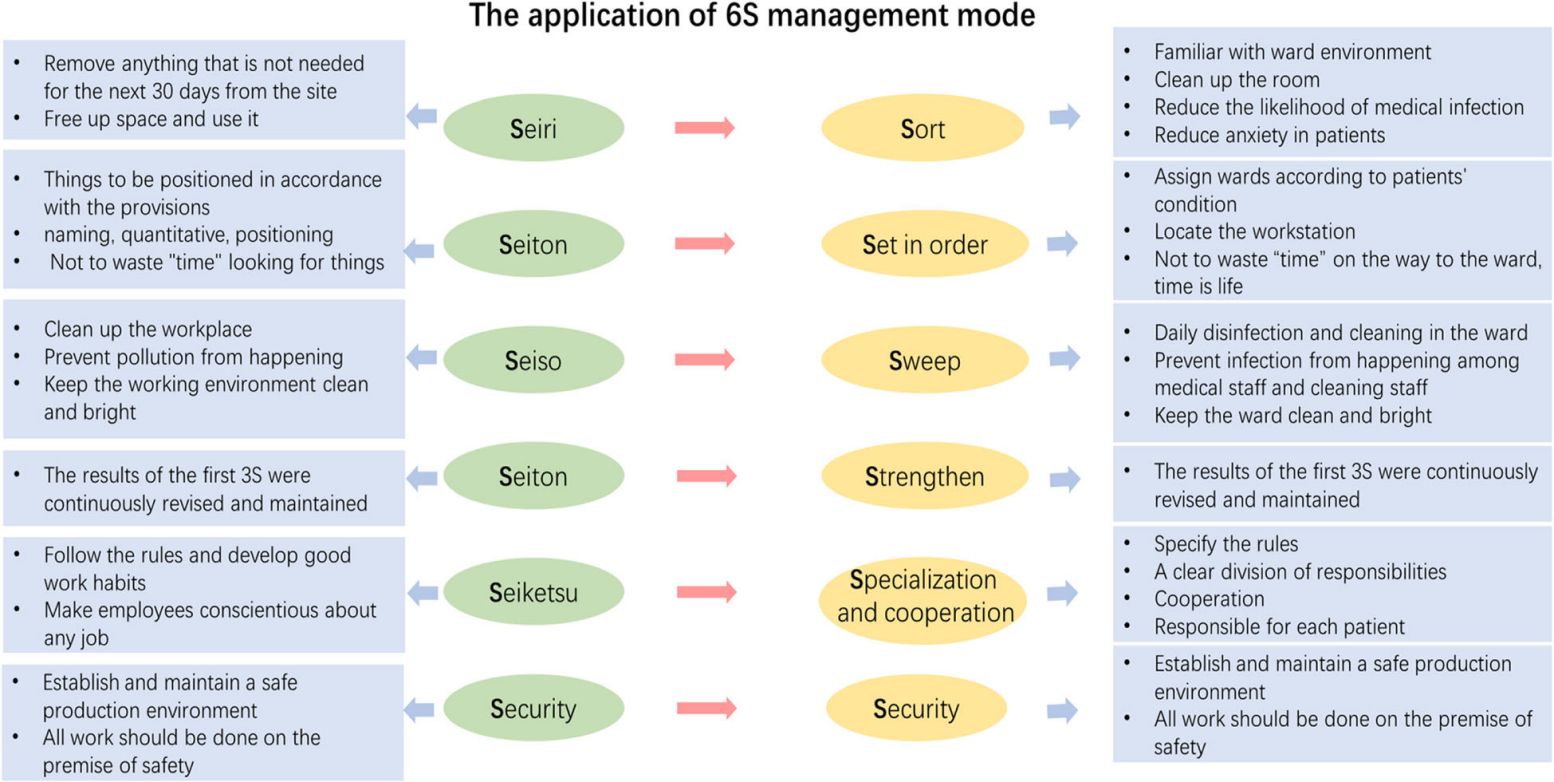
The application of 6S management mode

For the convenience of application and understanding, we transform the traditional management strategy 6S into Sort, Set in order, Sweep, Strengthen, Specialization and cooperation and Security. The specific applications are shown in Fig. 1.

### Sort

All relevant items were counted, sorted and rearranged. All items except for those immediately necessary in the ward were removed from the isolation area to create an adequate working space and a clean working environment. Moreover, a favourable ward environment also contributed to reducing the patients’ anxiety.

### Set in order

The original ward was reclassified into different areas, including normal living areas, semi-contaminated areas and isolation areas. In the isolation area, the workstation was located at the centre of the overall isolation ward and the beds were arranged so that the more severe patients were closer to the workstation.

### Sweep

Cleaning staff completed daily disinfection and cleaning in the ward as required (Q12h). Disposable contaminated items in the ward were collected, uniformly disposed of and transported in strict accordance with relevant regulations. Meanwhile, attention was paid to the disinfection of non-disposable protective equipment and instruments, the registration and verification of disinfection dates, and the integrity of the sealed packaging for the equipment after disinfection. During the working period, the cleaning personnel were supervised and assisted in performing personal protection activities.

### Strengthen

The sorting, reorganization and cleaning work were sustained, institutionalized and standardized. Through self-inspection and mutual supervision, the results of the first 3 model components were continuously revised and maintained.

### Specialization and cooperation

**Fig. 2:**
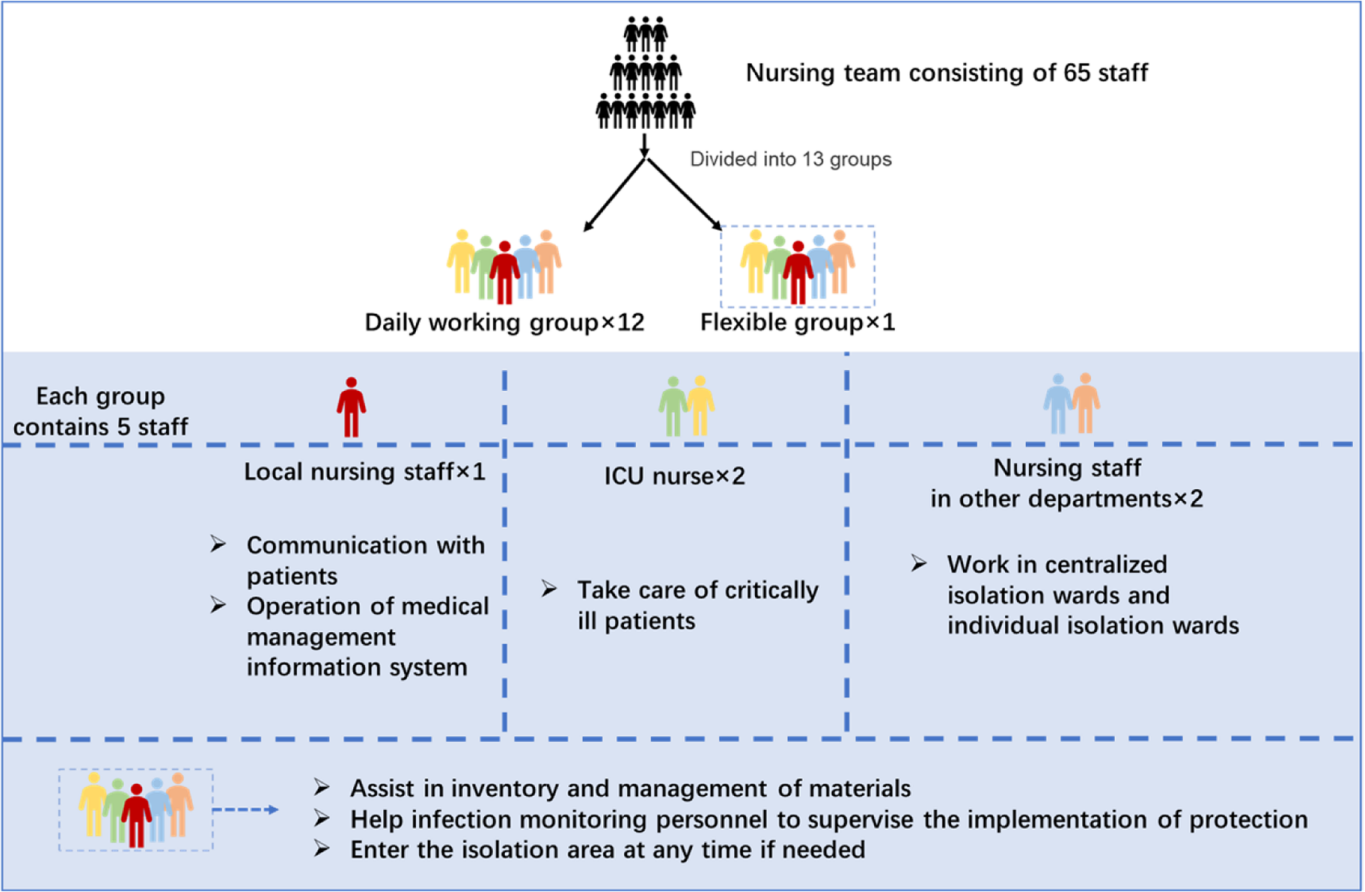
Groups of nursing staff and the division of labour

It was an unprecedented challenge for the nursing team, who came from multiple regions and disciplines and had different titles and levels of seniority, to complete the nursing work together. In the face of such a considerable challenge, the 65 nursing staff were divided into 13 nursing teams (including a flexible group), and a detailed division of labour was implemented (Fig. 2).

### Security

Throughout the process, the physical and psychological safety of all nursing staff was fully guaranteed. Each nursing staff group worked for 4 h. Additionally, the following measures were taken to provide psychological support and strengthen their ability to defend themselves against COVID-19:

1. Repeated training and assessment and continuous supervision, providing a sense of control.
2. Adequate protective materials.
3. Temperature measurements and physical examinations were performed twice a day, and abnormal situations were reported and addressed immediately.
4. The nursing team leader communicated with the team members in a timely manner to identify and solve problems as soon as possible.

## Application of the PDCA cycle to improve the quality of care

Overall, the management of the nursing team followed the PDCA (Plan-Do-Check-Act) cycle management principle as described in a previous study [5], thus enabling real-time planning, execution, inspection and improvement. During the working period, it was necessary to work together to improve team members’ work habits, mutual supervision and compliance with the work system so that the nursing team could perform their respective duties and maintain a good team spirit.

Through the abovementioned 6S and PDCA management modes, the following effects were achieved:

1. Medical staff and patients were confident in the way the epidemic was being managed.
2. The work efficiency was improved, and the risk of medical staff exposure was reduced.
3. The coordination within the team and the overall work efficiency were improved.
4. While still ensuring safety, the service life of instruments and equipment was maximized.
5. The goals of zero infections among staff, zero nursing accidents and zero complaints from patients were successfully achieved.

By combining 6S and PDCA management strategies with nursing work, we completed a ward renovation and nursing team integration in a short time and implemented epidemic prevention work quickly and efficiently. Hopefully, our experience can be helpful for anti-epidemic work abroad.

## Data Availability

All data generated or analysed during this study are included in this published article.
